# Gene Family Analysis of the *Arabidopsis NF-YA* Transcription Factors Reveals Opposing Abscisic Acid Responses During Seed Germination

**DOI:** 10.1007/s11105-014-0704-6

**Published:** 2014-02-25

**Authors:** Chamindika L. Siriwardana, Roderick W. Kumimoto, Daniel S. Jones, Ben F. Holt

**Affiliations:** Department of Microbiology and Plant Biology, University of Oklahoma, 770 Van Vleet Oval, GLCH, Room 43, Norman, OK 73019 USA

**Keywords:** Germination, Abscisic acid, Signaling, Transcription factor, Plant hormone, NUCLEAR FACTOR-Y

## Abstract

In the plant kingdom, each of the *NUCLEAR FACTOR-Y* (*NF-Y*) transcription factor families, *NF-YA*, *NF-YB*, and *NF-YC*, has undergone a great expansion compared to the animal kingdom. For example, *Arabidopsis thaliana* has 10 members of each gene family compared to only one in humans. Progress towards understanding the significance of this expansion is limited due to a lack of studies looking at the complete gene family during plant development. In the current study, transgenic overexpression lines were created for all 10 *Arabidopsis NF-YA* genes and examined for general development and alterations in abscisic acid (ABA)-mediated seed germination. *NF-YA* overexpression typically led to severe growth retardation and developmental defects, which extended from embryogenesis through to adult plants. Although overexpression of all *NF-YA* family members consistently led to growth retardation, some transgenic lines were hypersensitive to ABA during germination while others were hyposensitive. The opposing germination phenotypes were associated with the phylogenetic relationships between the *NF-YA* members. In addition, ABA marker genes were misregulated and ABA induction of gene expression was reduced in the overexpressors. Collectively, this study demonstrates that although NF-Ys have retained high degrees of similarity, they have evolved unique and sometimes opposing roles during plant development.

**Electronic supplementary material:**

The online version of this article (doi:10.1007/s11105-014-0704-6) contains supplementary material, which is available to authorized users.

## Introduction

NUCLEAR FACTOR-Y (NF-Y) transcription factors bind DNA as complexes composed of three unique subunits, called NF-YA, NF-YB, and NF-YC. While common throughout the eukaryotic lineage, the three NF-Y families have undergone an expansion in plants, with most species encoding ~10 genes for each family (Gusmaroli et al. 2001, 2002; Stephenson et al. 2007; Thirumurugan et al. 2008; Siefers et al. 2009; Cao et al. 2011; Petroni et al. 2012; Laloum et al. 2013). *A. thaliana* (*Arabidopsis*) has 10 *NF-YA*, 10 *NF-YB*, and 10 *NF-YC*, and since NF-Y binds to DNA as a heterotrimer, this leads to the possibility of 1,000 unique NF-Y transcription factors (Petroni et al. 2012). The large number of possible complexes suggests the potential to regulate diverse plant processes. NF-Ys have demonstrated roles in abscisic acid (ABA) responses (Nelson et al. 2007; Warpeha et al. 2007; Li et al. 2008; Yamamoto et al. 2009; Leyva-Gonzalez et al. 2012; Kumimoto et al. 2013; Mu et al. 2013), photoperiod-dependent flowering (Ben-Naim et al. 2006; Wenkel et al. 2006; Cai et al. 2007; Chen et al. 2007; Kumimoto et al. 2008, 2010), embryogenesis (West et al. 1994; Lotan et al. 1998; Kwong et al. 2003; Lee et al. 2003), endoplasmic reticulum stress responses (Liu and Howell 2010), salt stress responses (Li et al. 2013), photosynthesis (Kusnetsov et al. 1999; Stephenson et al. 2010), root elongation (Ballif et al. 2011), and nodule development (Combier et al. 2006, 2008; Zanetti et al. 2010).

NF-Y family proteins have retained a high degree of similarity, especially in the residues necessary for complex formation and DNA binding; therefore, how NF-Ys have diverged to regulate a diverse set of development processes is still in question (Siefers et al. 2009; Laloum et al. 2013). NF-YA proteins are typified by a 53-amino acid conserved domain which makes physical contacts with DNA at *CCAAT* box *cis*-elements and mediates interactions with the NF-YB/NF-YC dimer (Olesen and Guarente 1990; Maity et al. 1992; Xing et al. 1993, 1994; Nardini et al. 2013). While the NF-YB and NF-YC subunits are abundant in vivo, NF-YA is limiting for trimer formation and subsequent DNA binding (Dolfini et al. 2012). A combination of animal and plant literature demonstrated that the expression of NF-YA subunits is highly regulated at the transcriptional, posttranscriptional, and posttranslational level. At the transcriptional level, tissue-specific expression of the expanded *NF-YA* gene family in plants has shown spatial and temporal specialization (Stephenson et al. 2007; Siefers et al. 2009; Cao et al. 2011). In animals, NF-YA protein is targeted for ubiquitination and subsequently degraded by proteasome (Manni et al. 2008). Due to the high conservation of the residues targeted for ubiquitination, this likely also holds true for plant NF-YAs. In addition, plant *NF-YA* transcripts are targeted by a family of microRNAs called *miR169* (Rhoades et al. 2002). In turn, *miR169* abundance is regulated by the important stress hormone ABA.

Several recent publications have demonstrated that NF-YA subunits play an essential role during ABA-mediated responses in plants. ABA signals are perceived via the PYRABACTIN RESISTANCE1 (PYR1)/PYR1-LIKE (PYL)/REGULATORY COMPONENTS OF ABA RECEPTOR (RCAR) family of soluble receptors (Fujii et al. 2009; Ma et al. 2009; Melcher et al. 2009; Miyazono et al. 2009; Nishimura et al. 2009; Park et al. 2009; Santiago et al. 2009). Once ABA is bound to PYR/PYL, a signaling cascade is initiated through PP2C phosphatases and SnRK2 kinases to activate basic leucine zipper (bZIP) transcription factors that bind ABA response elements (ABREs) within the promoters of ABA response genes (Gosti et al. 1999; Merlot et al. 2001; Saez et al. 2004; Choi et al. 2005; Finkelstein et al. 2005; Furihata et al. 2006; Fujii and Zhu 2009; Rubio et al. 2009; Umezawa et al. 2009; Vlad et al. 2009; Yoshida et al. 2010). Select NF-YA subunits were shown to regulate the expression of these core ABA signaling components (Leyva-Gonzalez et al. 2012). Microarray analysis of *Arabidopsis NF-YA2*, *NF-YA3*, *NF-YA7*, and *NF-YA10* driven by an inducible promoter revealed that transcript levels of several *PYR/PYL/RCAR*, *PP2C*, and *SnRK2* family members were consistently downregulated.

In addition to regulating ABA signaling components, mutants and overexpressors of *NF-YAs* have ABA-related developmental phenotypes during drought responses and seed germination. *NF-YA5* transcripts increase in response to drought in an ABA-dependent manner (Li et al. 2008). The increase in transcript of *NF-YA5* is attributed to drought-induced downregulation of *miR169a*, which targets *NF-YA5* transcripts for degradation. Further, plants overexpressing *NF-YA5* were drought tolerant, whereas mutants were susceptible. Two recent publications further demonstrated that overexpression of selected members of the *NF-YA* family leads to ABA-mediated seed germination phenotypes. Briefly, qualitative analyses demonstrated that overexpression of *NF-YA1*, *NF-YA2*, *NF-YA3*, *NF-YA7*, *NF-YA9*, and *NF-YA10* led to ABA hypersensitivity (Leyva-Gonzalez et al. 2012; Mu et al. 2013). In addition, *NF-YA5* mutants were hypersensitive to ABA during seed germination (Warpeha et al. 2007). NF-YC subunits were also recently shown to be involved in ABA responses. Interestingly, different NF-YC subunits can have unique and opposing functions in ABA-mediated seed germination (Kumimoto et al. 2013). Mutants of *NF-YC4* were hypersensitive to ABA, whereas mutants of *NF-YC3* and *NF-YC9* were hyposensitive to ABA during germination. The presence of opposing germination phenotypes in *NF-YC* mutants indicated that *NF-YA*s might also be involved in similar phenomena; however, this had not been systematically examined for the entire family.

Here, we present a complete family analysis of the *Arabidopsis NF-YA*. All 10 *Arabidopsis NF-YA* genes were systematically overexpressed, and the resulting phenotypes were characterized relative to morphological development and ABA-mediated germination. Due to the presence of 10 *NF-YA* genes with high levels of amino acid similarity and extensive overlap in tissue-specific expression patterns (Siefers et al. 2009), we reasoned that overexpression would be a more fruitful first approach. Additionally, loss-of-function mutants in *NF-YA1* and *NF-YA2* are lethal (Pagnussat et al. 2005; Meinke et al. 2008). Overexpression of all *NF-YA* led to severe growth retardation, which was seen from embryo development through the adult plant. Although all overexpressors showed various levels of growth retardation, some transgenic lines were hypersensitive and others were hyposensitive to germination on ABA. ABA marker genes were misregulated, and the ability of exogenously applied ABA to induce transcription of marker genes was attenuated in the overexpressors. The opposing ABA phenotypes were associated with phylogenetic relationships between the *NF-YAs*, indicating that members of this closely related gene family evolved distinct roles during ABA-mediated seed germination.

## Materials and Methods

### Phylogenetic Analysis

Full-length cDNA sequences for the coding regions of *NF-YA* subunits were obtained from TAIR (http://www.arabidopsis.org (Huala et al. 2001)). Phylogenetic analyses were conducted in MEGA5 (Tamura et al. 2011). The maximum parsimony method was used to infer evolutionary history as described previously (Felsenstein 1985; Nei 2000). The phylogenetic tree is drawn to scale.

### Construction of Transgenic Lines

The full-length coding region of each *NF-YA* gene (*NF-YA1* to *NF-YA9*) was amplified from cDNA by PCR using Pfu Ultra II (cat#600670, Agilent Technologies) and ligated into the Gateway® entry vector pENTR/D-TOPO (cat#45-0218, Invitrogen). All constructs were sequenced and found to be identical to sequences at The *Arabidopsis* Information Resource (http://www.arabidopsis.org (Huala et al. 2001)). *NF-YA10* cDNA in pDONR221 was obtained from ATOME1 ORFEOME library (stock#51B10, CNRGV). All *NF-YA* cDNA clones were introduced to the plant expression destination vector pEarlyGate102 (stock#CD3-684, ABRC) (Earley et al. 2006) using the Gateway® LR Clonase II™ reaction kit (cat#56485, Invitrogen). The 35S cauliflower mosaic virus promoter (p35S) (Kay et al. 1987) was driving the expression of each gene. Transgenic plants were generated using agrobacterium-mediated floral dipping described in previous studies (Clough and Bent 1998). At least two independent homozygous or hemizygous transgenic lines were examined for each *NF-YA* (Table S1).

### Plant Cultivation and Germination Assays

*A. thaliana* ecotype Columbia (Col-0) was used as the wild type for all experiments. For morphological studies and generation of matched seed sets, plants were grown in standard long-day conditions (16-h light/8-h dark cycle) in a custom walk-in chamber. Plant growth medium contained equal parts Farfard C2 and Metromix 200 (17,620 cm^3^ total soil mixture) supplemented with 40 g MARATHON pesticide and 3,785 cm^3^ distilled water with Peter’s fertilizer (NPK 20:20:20). Plants were watered with dilute Peter’s fertilizer (at 1/10 recommended feeding levels) throughout the growth cycle. For western blot, germination assays, qPCR, and microscopy seed plates were cold-stratified in the dark for 48 h and placed in a Conviron ATC13 growth chamber at 22 °C with continuous light.

Germination assays were always performed on matched seed sets that were after ripened for 4 months. Seeds were sterilized by treating with 70 % ethanol for 5 min and 50 % household bleach for 5 min followed by five washes of sterile distilled water. Seeds were germinated on Gamborg’s B5 media or B5 supplemented with (+)-ABA (cat#A4906, Sigma). Germination was scored as the emergence of the radical tip from the seed coat (Bewley 1997). These experiments were done in triplicate (*n* = 3), with a total of at least 80 seeds used per genotype and repeated three times with independent, matched seed sets with the same results. For the statistical analysis, the observed frequencies were compared with expected frequencies with Fisher’s exact tests as previously described within INSTAT (GraphPad Software, La Jolla, CA) (Kumimoto et al. 2013).

### Microscopy

*p35S::NF-YA5* and *pNF-YA::GUS/GFP* lines (Siefers et al. 2009) were imaged with a Zeiss Axio Imager.Z1/ApoTome microscope (Carl Zeiss). Prior to imaging, *pNF-YA:GUS/GFP* seed coats (including endosperm) and embryos were stained by placing in beta-glucuronidase (GUS) staining solution and incubated overnight at 37 °C in the dark (Perry and Wang 2003). Subcellular localization was determined in 4-day-old seedlings counterstained by incubating in 50 μg/mL propidium iodide (PI) for 5 min, followed by washing in deionized (DI) water for 5 min. Seedlings were mounted in DI water, and roots were imaged using a Leica TCS SP8 confocal laser scanning microscope with a ×40 water immersion objective. Sequential scanning mode was used for cyan fluorescence protein (CFP) and PI detection where CFP was excited using 458 nm laser with emission detected at 462–536 nm and PI was excited using a 561-nm laser with emission detected at 582–673 nm. Approximately 200 serial sections of root tip were imaged with an average cubic voxel size of 190 × 190 × 190 nm starting with the root epidermis closest to the coverslip imaging through to the stele. For DNA labeling, tissue was fixed in 4 % PFA in PBS for 2 min incubated in 5 μg/mL Hoechst 33342 for 50 min, mounted on DI water, and excited with a 405-nm laser. Images were processed using ImageJ 1.46r (http://rsb.info.nih.gov/ij/) (Schneider et al. 2012) where average intensities of both CFP and PI channels through the series were taken and merged.

### Western Blot

Total protein was extracted from 14-day-old plants by grinding in lysis buffer (20 mM Tris, pH 8.0, 150 mM NaCl, 1 mM EDTA, pH 8.0, 1 % Triton X-100, 1 % SDS with fresh 5 mM DTT, and 100 μM MG132). NF-YA-CFP/HA was probed with high-affinity anti-HA primary antibody (cat#11 867 423 001, Roche) and goat anti-rat secondary antibody (cat#SC-2032, Santa Cruz Biotechnology). The Bio-Rad ChemiDoc XRS imaging system was used for visualizing the protein blot after incubations with ECL plus reagent (cat#RPN2132, GE Healthcare). Equivalent loading and transfer efficiency was determined by staining the protein blot with Ponceau S (cat#P3504, Sigma-Aldrich).

### qPCR Analysis

Matched seed sets were germinated on Gamborg’s B5 medium, with or without 1 μM (+)-ABA. Total seed RNA was extracted using the E.Z.N.A. Plant RNA Kit (cat#R6827-01, Omega Bio-Tek) according to the manufacturer’s instructions for difficult samples. Genomic DNA was digested during RNA extraction by treating the columns with DNase (cat#E1091. Omega Bio-Tek). First-strand cDNA was synthesized using the SuperScript III First-Strand Synthesis System (cat#18080-051, Invitrogen). qPCR was performed using a CFX Connect™ Real-Time PCR Detection System (Bio-Rad) with the SYBR Green qPCR Master Mix (cat#K0222, Fermentas). Gene expression analysis was done using the CFX Manager™ software (Bio-Rad). Normalized expression, ∆∆*C*_q_, was selected as the analysis mode. Samples were normalized to a constitutively expressed reference gene, At2g32170 (Czechowski et al. 2005). Three biological replicates were used for the qPCR, which was repeated three times with the same results. Statistical analysis was done with two-way ANOVA (*P* < 0.05), in which genotype and seed growth media were used as the two variables, followed by Bonferroni multiple comparisons post hoc test against Col-0 on B5 media or on B5+1 μM ABA (Gutierrez et al. 2008; Rieu and Powers 2009). Primer sequences are available upon request.

## Results

### The *Arabidopsis NF-YA* Family Clusters into Five Groups of Paralogs

**Fig. 1:**
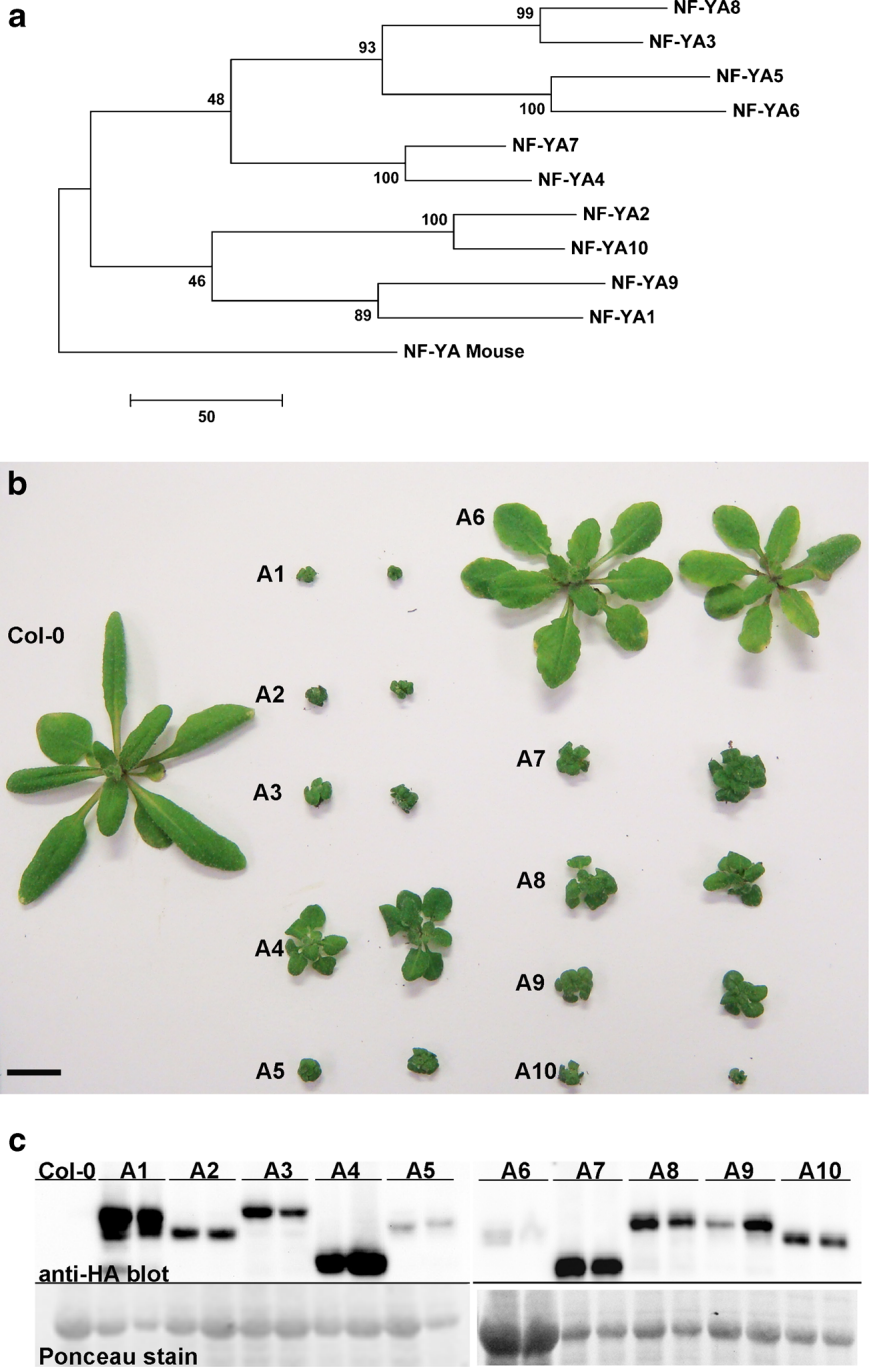
Characterization of *p35S:: NF-YA* lines. **a** Phylogenetic relationship between the *Arabidopsis NF-YA* genes. **b** Phenotypes for two independent lines of 3-week-old *p35S::NF-YA* plants compared to Col-0. **c** Protein blot for the two independent transgenic lines of each *p35S::NF-YA*

Phylogenetic analyses showed that the 10 members of the *NF-YA* gene family cluster into five groups of apparent paralogs: *NF-YA1/NF-YA9*, *NF-YA2/NF-YA10*, *NF-YA3/NF-YA8*, *NF-YA4/NF-YA7*, and *NF-YA5/NF-YA6* (Fig. 1a). Although the amino acid sequence of the NF-YA core domain is highly conserved, there are a few amino acids that are unique, especially in the early diverging paralogs *NF-YA1/NF-YA9* and *NF-YA2/NF-YA10* (Fig. S1). The NF-YA subunits diverge outside the core domain; however, the pairs of paralogs maintain high identity throughout the amino acid sequence (Figs. S1 and S2). The combination of highly conserved core domains and diverging, non-conserved regions suggested that studying overexpressors of the complete *NF-YA* gene family would potentially reveal both common and unique phenotypes.

### Overexpression of *NF-YAs* Causes Severe Growth Retardation

To characterize the developmental phenotypes associated with *NF-YA* overexpression, qualitative and quantitative analyses were performed on two independent transgenic lines for each gene. Most *NF-YA* overexpression lines were shorter with smaller rosette diameters and produced fewer, smaller siliques than wild-type plants (Figs. 1b and S3). The only exceptions were *p35S::NF-YA4* and *p35S::NF-YA6*, where one or both plant lines were similar to the wild type. Although most *p35S::NF-YA* plants exhibited varying levels of dwarfism, they all went through the same developmental stages as wild-type plants with only moderate delays. Plant lines used for analysis had demonstrated accumulation of the transgenic proteins (Fig. 1c). The level of protein expressed varied with *p35S::NF-YA1* and *p35S::NF-YA4* having the strongest expression and *p35S::NF-YA6* the weakest. The phenotypes seen here are in agreement with those in previous reports showing that overexpression of a smaller subset, *NF-YA2*, *NF-YA4*, *NF-YA7*, and *NF-YA10*, also led to dwarf phenotypes (Liu and Howell 2010; Leyva-Gonzalez et al. 2012).

### *p35S:*:*NF-YA5* and *p35S::NF-YA6* Produce Cotyledon-Like Leaves

**Fig. 2:**
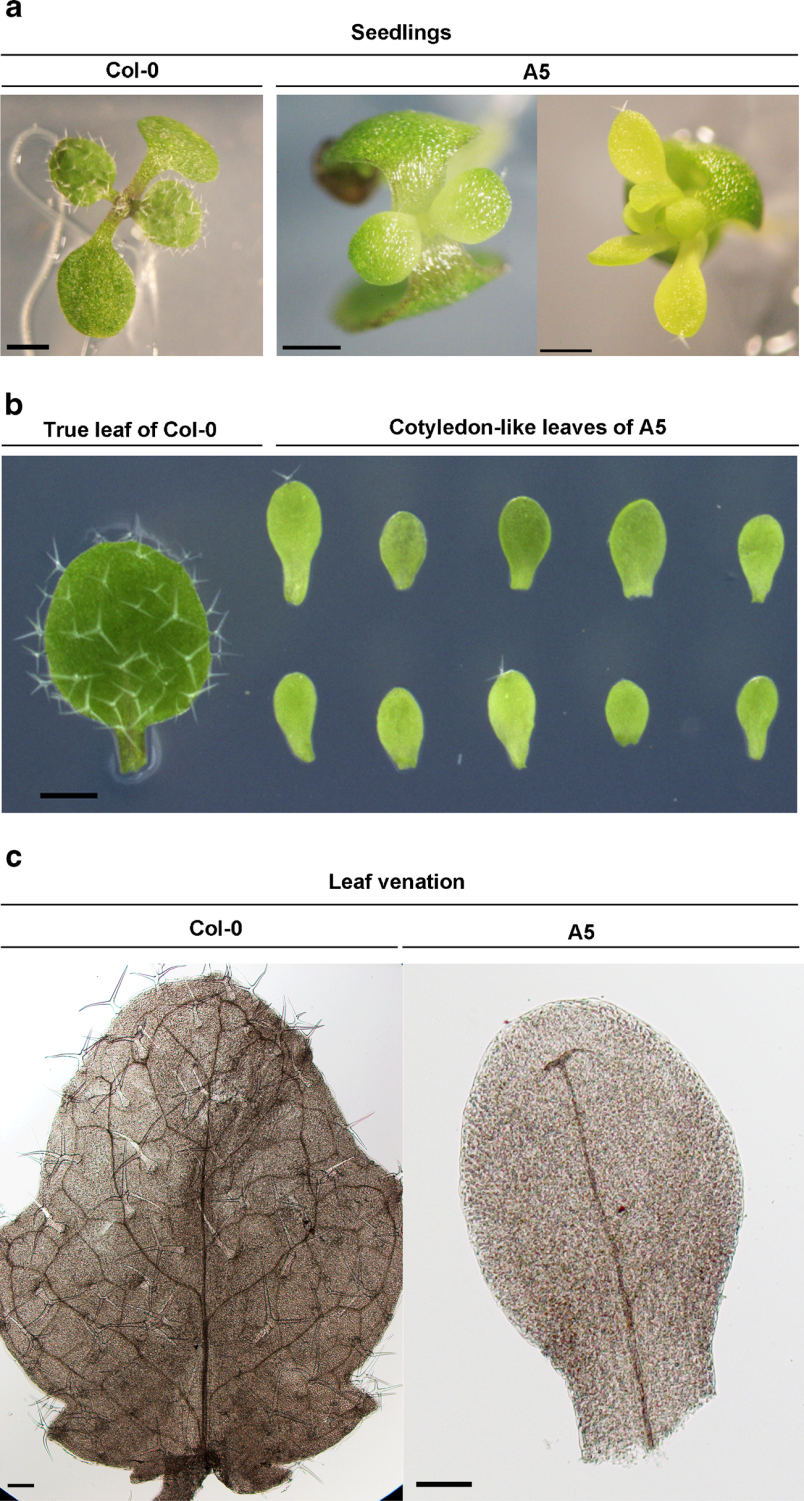
*p35S::NF-YA5* seedlings develop cotyledon-like leaves. **a** Seedlings of Col-0 and *p35S:: NF-YA5.* **b** True leaves of Col-0 in comparison to cotyledon-like leaves of *p35S::NF-YA5*. **c** Differential interference contrast (DIC) microscopy images of leaf venation in Col-0 and *p35S::NF-YA5*. The *scale bar* in **a** equals 1 mm (for Col-0) and 0.5 mm (for *p35S::NF-YA5*) **b** equals 2 mm, and **c** equals 50 μm

Two *NF-YB* subunits, LEAFY COTYLEDON 1 (LEC1/NF-YB9) and LEC1-LIKE (L1L/NF-YB6), are essential for embryo development (West et al. 1994; Lotan et al. 1998; Kwong et al. 2003; Lee et al. 2003; Junker et al. 2012). Mutants of *LEC1* and *LEC1-L* produce cotyledons with leaflike characters (e.g., trichomes), whereas overexpressors can produce cotyledon-like leaves. Although *NF-YBs* required for embryo development have been identified, the presumed *NF-YA* and *NF-YC* remained unidentified. Recently, Mu et al. (2013) published that overexpressors of *NF-YA1*, *NF-YA5*, *NF-YA6*, and *NF-YA9* produce cotyledon-like leaves. Examining all 10 *NF-YA* overexpression lines, we found that this phenotype occurs somewhat rarely and inconsistently for most lines. The exceptions were the paralogous *p35S::NF-YA5* and *p35S::NF-YA6* lines where we consistently observed cotyledon-like leaves in the normal position of the first set of true leaves (Fig. 2a). This phenotype often persisted for multiple pairs of leaves in *p35S::NF-YA5* plants and ultimately precluded seed set and further characterization of *p35S::NF-YA6*. In addition to gross morphological appearance resembling elongated cotyledons, leaves that should have developmentally corresponded to the first non-embryonic, true leaves were considerably smaller, had less chlorophyll, and typically lacked or had very few trichomes relative to wild-type controls (Figs. 2b and S4). The cotyledon-like leaves of *p35S::NF-YA5* were further observed by differential interference contrast (DIC) microscopy and found to have vascular defects, including vascular tissue that was largely limited to the midrib region (Fig. 2c). Although the *p35S::NF-YA5* seedlings had severe growth defects, they were tolerant to salt and osmotic stress (Fig. S5).

### NF-YA Proteins Are Localized to the Nucleus

Studies in animal systems have shown that the NF-YA subunit is primarily localized to the nucleus (Frontini et al. 2004; Kahle et al. 2005). The high degree of conservation between plant and animal NF-Ys (Siefers et al. 2009) suggested that a similar localization pattern would be seen in plants. Supporting this argument, the positively charged arginine and lysine residues in the core domain of the human NF-YA subunit that are required for nuclear localization (Kahle et al. 2005) are highly conserved in *Arabidopsis* (Fig. S1) (Siefers et al. 2009).

**Fig. 3:**
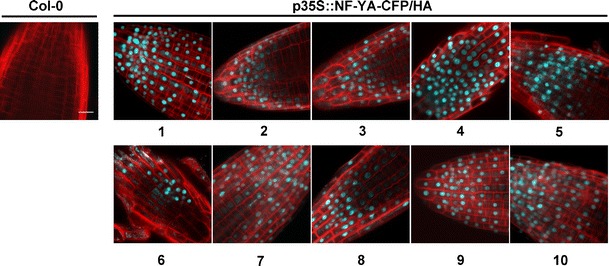
*NF-YA* proteins are nuclear-localized. Protein localization in Col-0 and *p35S::NF-YA-CFP/HA* overexpression lines (numbers below pictures represent the individual NF-YA genes). The cyan fluorescence protein (CFP) signal (*blue*) was always strongly associated with the nucleus (note that localization was confirmed by merged images, combining the CFP localization of *NF-YAs* with DIC imaging and Hoechst 33342 labeling staining of the nucleus—Fig. S6). The cell walls are stained with propidium iodide (*red*). The *scale bar* in Col-0 equals 15 μm

Localization of all 10 NF-YA-CFP/HA proteins was studied using confocal microscopy. The CFP signal was always strongly associated with the nucleus (Fig. 3). The strength of the CFP signal corresponded well with the level of protein expression seen on the western blot (Fig. 1c). The strongest expressing lines, *p35S::NF-YA1* and *p35S::NF-YA4*, had the strongest CFP signal, whereas the weakest expressing line, *p35S::NF-YA6*, had the weakest signal. This data supports and extends previously published data showing that *Arabidopsis* NF-YA1, NF-YA4, and NF-YA5 are nuclear localized (Li et al. 2008; Liu and Howell 2010; Li et al. 2013).

### *p35S::NF-YAs* Have Opposing Germination Phenotypes on ABA

*NF-YC* mutants can have opposing germination phenotypes on ABA (Kumimoto et al. 2013). Since the NF-Y complex binds DNA as a trimer (Sinha et al. 1996; Romier et al. 2003; Nardini et al. 2013), we reasoned that this was likely to hold true for the NF-YA and concurrently tested all 10 subunits in this study. For ease of comparison, results were graphed based on phylogenetic relationships (Fig. 1a), with the apparent closest paralogs placed on the same graph in each instance.

**Fig. 4:**
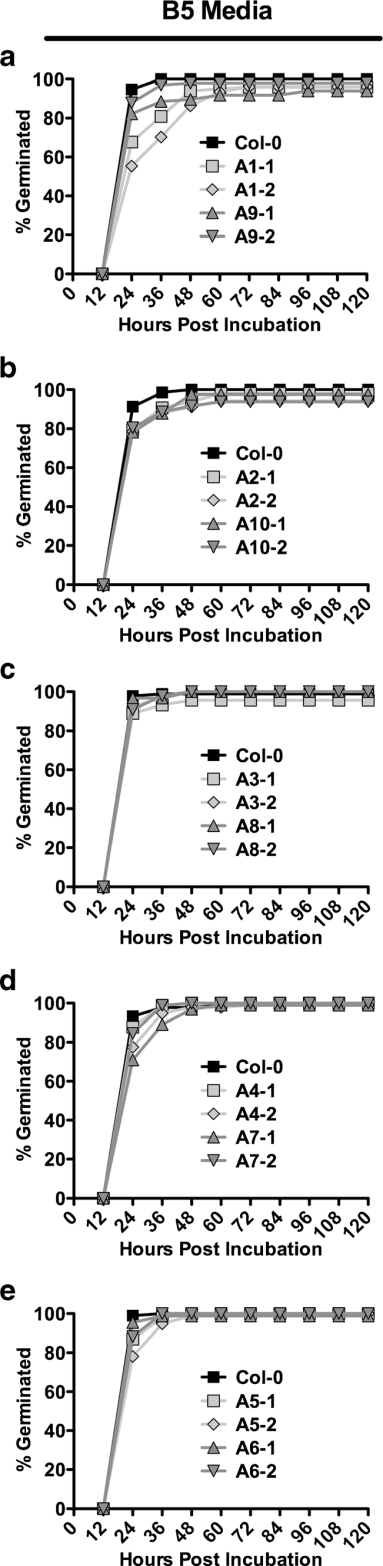
Seed germination on Gamborg’s B5 medium for *p35S::NF-YA*. **a**–**e** Germination curves for two independent lines each of *p35S::NF-YA* overexpressors compared to Col-0

**Fig. 5:**
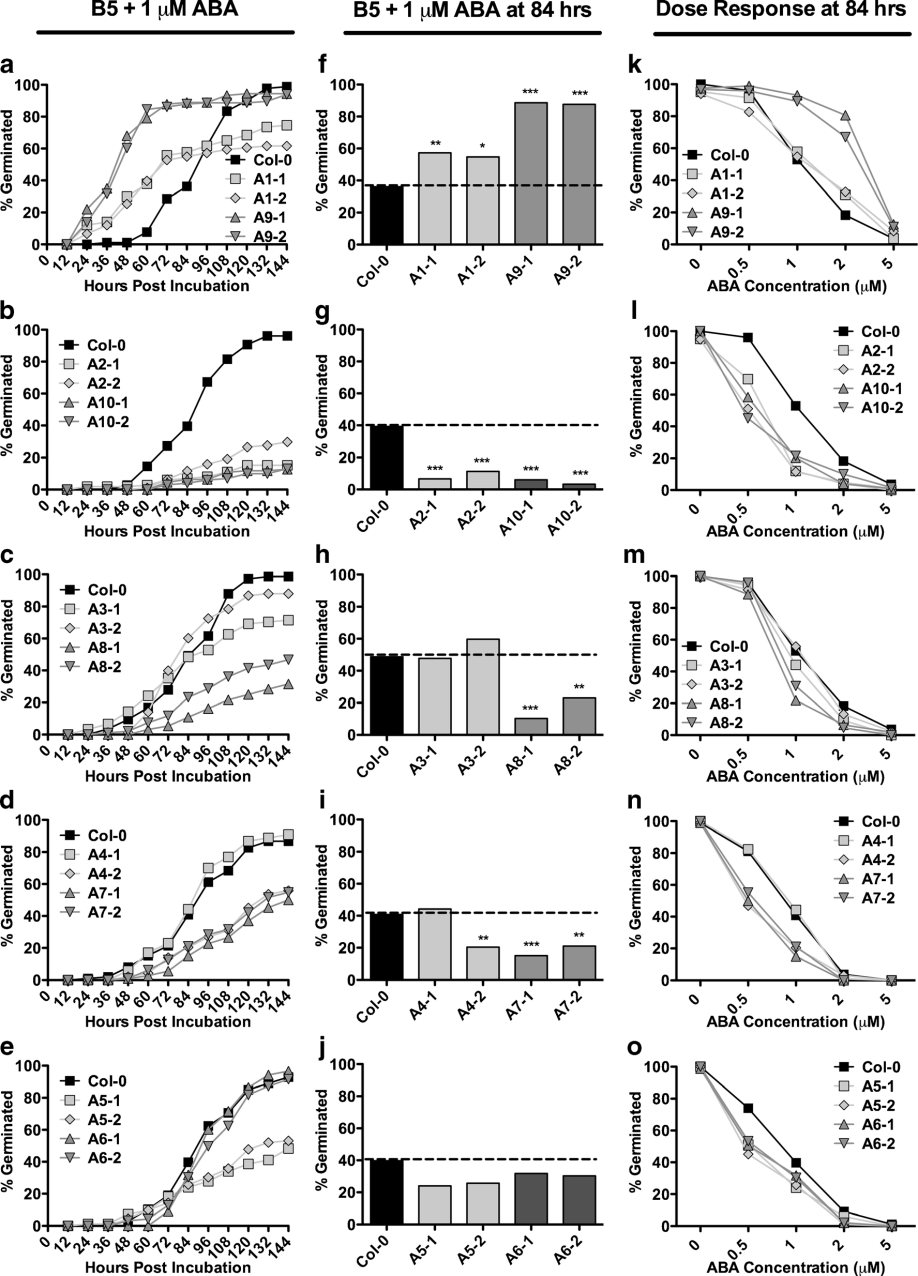
*p35S::NF-YA* overexpressors show opposing germination phenotypes on ABA. **a**–**e** Germination curves for two independent *p35S::NF-YA* lines. **f**–**j** Germination at 84 h postincubation. **k**–**o** Dose responses on 0.5, 1, 2, and 5 μM ABA at 84 h postincubation. *Asterisks* for **f** to **j** are Fisher’s exact test *p* values; *p < 0.01, **p < 0.001, ***p < 0.0001

On non-ABA media (Gamborg’s B5), most *p35S::NF-YA* lines germinated similarly to parental Col-0, although some lines showed minor delays (Fig. 4a–e). Nevertheless, all plant lines reached ∼50 % germination by 18–24 h postincubation and ∼100 % germination by 48 h postincubation. On media supplemented with 1 μM (+)-ABA, germination of parental Col-0 was delayed by approximately 72 h. Conversely, *NF-YA* overexpression caused highly variable responses to ABA (Fig. 5a–e). Most interestingly, we found that overexpression of the closely related (Fig. 1a) *NF-YA1* and *NF-YA9* genes resulted in early germination; *p35S::NF-YA1* lines reached 50 % germination ∼20 h earlier than parental Col-0, while *p35S::NF-YA9* lines germinated a full 48 h earlier (Fig. 5a). In contrast, overexpression of *NF-YA2*, *NF-YA4*, *NF-YA7*, *NF-YA8*, and *NF-YA10* resulted in late germination. To statistically confirm the apparent differences from parental Col-0, we performed Fisher’s exact tests at 84 h postincubation (Fig. 5f–j, 84 h was chosen because it is equivalent to ∼50 % germination for Col-0 in most experiments). Additionally, we examined dose–response curves for each transgenic line using the 84-h time point (Fig. 5k–o). Collectively this data demonstrates that *NF-YA* overexpression consistently alters ABA responses but that *NF-YA*s can cause opposing phenotypes in response to ABA. Additionally, we note that the ABA phenotypes are not directly correlated in any obvious way with the gross morphological data reported above (i.e., dwarf plants can give rise to both ABA-susceptible and ABA-resistant seeds, depending on the overexpressed *NF-YA* gene).

To see if the ABA responses of *p35S::NF-YA* are developmentally stage dependent (i.e., if ABA responses extend beyond germination), the effect of ABA on root elongation was tested. Four-day-old seedlings of selected *p35S::NF-YA* plant lines were initially grown on non-ABA media and then transferred to non-ABA (control) or ABA media (5 μM (+)-ABA). We selected *p35S::NF-YA7*, *p35S::NF-YA8*, and *p35S::NF-YA9* because they were relatively healthy (i.e., the dwarf stature was not as severe as other stable lines) and had opposing phenotypes in the germination assays (Fig. 5). On non-ABA media, primary root lengths were shorter for all *p35S::NF-YA* lines compared to wild-type plants. Because of these differences in primary root elongation, results were graphed as the percent root elongation compared to non-ABA media. The results showed that the primary root growth of all three lines was hypersensitive to ABA (Fig. S7). Thus, in contrast to the opposing germination phenotypes, all three *p35S::NF-YA* lines showed the same negative effect of *NF-YA* overexpression during root growth on ABA media.

### *NF-YA* Genes Are Expressed in Embryos and the Endosperm

**Fig. 6:**
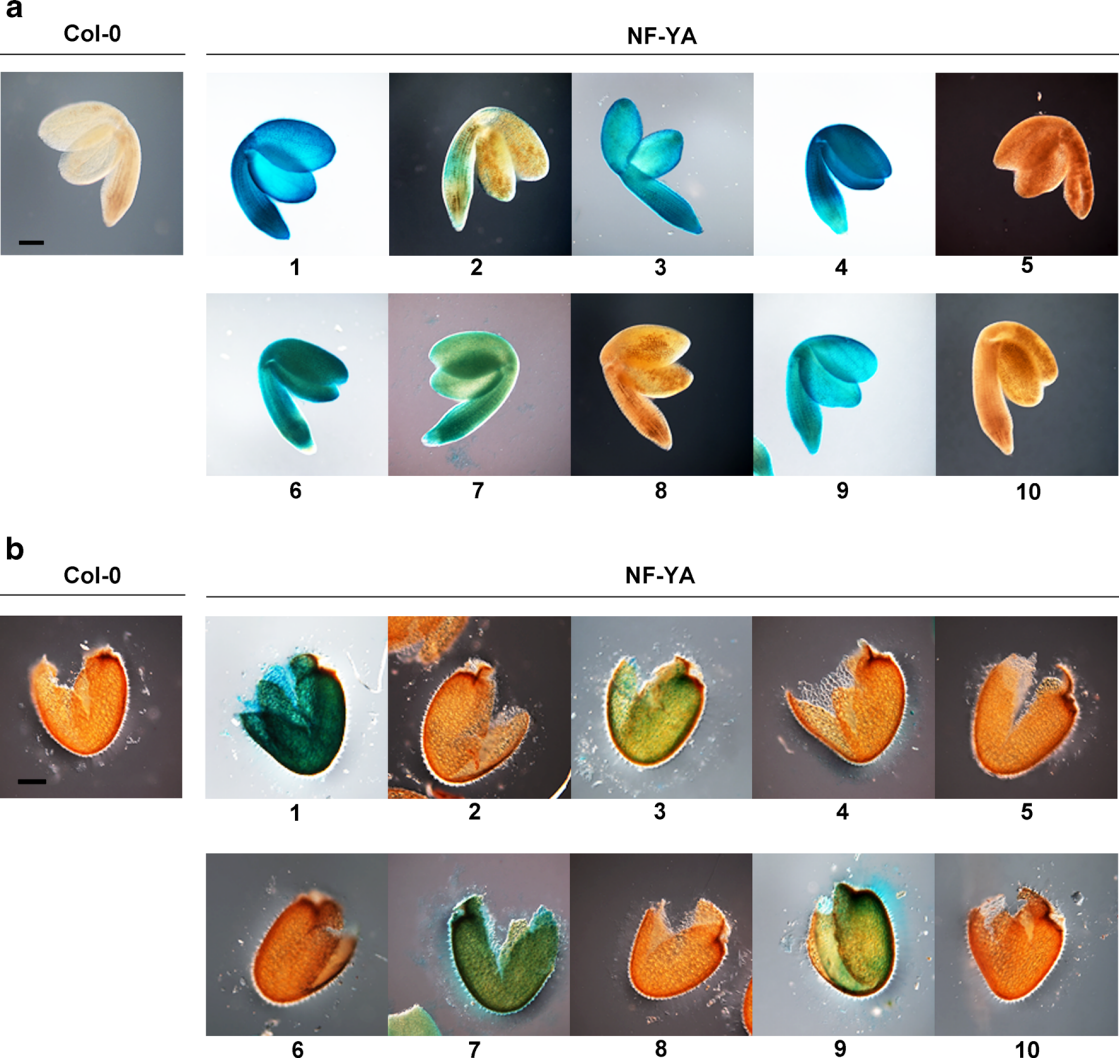
*NF-YA* genes are expressed in the endosperm and embryos (numbers below pictures represent the individual NF-YA genes). The native expression of *NF-YA* genes in seeds imbibed for 24 h are shown for **a** embryos and **b** seed coat/endosperm. The *scale bar* in Col-0 equals 50 μm

Most of the *NF-YA* genes showed ABA-related germination phenotypes when overexpressed with the p35S promoter. However, a disadvantage with using overexpression constructs is that genes that do not have a biological role in a tissue may show a phenotype due to ectopic overexpression. To determine which *NF-YA* genes are likely to have a native biological role during seed germination, transgenic plants expressing the *NF-YAs* fused to the GUS reporter gene and driven by their native promoter were examined (Siefers et al. 2009). This analysis showed that *NF-YA1*, *NF-YA2*, *NF-YA3*, *NF-YA4*, *NF-YA6*, *NF-YA7*, *NF-YA8*, and *NF-YA9* were expressed in embryos and *NF-YA1*, *NF-YA2*, *NF-YA3*, *NF-YA7*, and *NF-YA9* were expressed in the endosperm (Fig. 6). The only genes that did not show expression in the embryo or the endosperm were *NF-YA5* and *NF-YA10*. We compared these findings to publicly available expression data (*Arabidopsis* e-FP browser, http://bar.utoronto.ca/efp/cgi-bin/efpWeb.cgi) (Winter et al. 2007) and found similar results (Fig. S8). The only significant difference was *NF-YA8* where we measured fairly weak expression, but publicly available data suggested a moderately strong expression. Collectively, our data supports likely roles for most members of this gene family during seed development.

### ABA-Related Genes Show Altered Expression in *p35S::NF-YA*

To further examine how *NF-YA* genes regulate ABA responses during seed germination, the expression of various ABA-related markers was examined. This included genes involved in ABA biosynthesis and catabolism, ABA signaling, and various downstream responses. *p35S::NF-YA8* and *p35S::NF-YA9* were selected for qPCR analyses due to their opposing germination phenotypes (*p35S::NF-YA8* is hypersensitive and *p35S::NF-YA9* is hyposensitive to ABA, Fig. 5a, b). *NF-YA8* and *NF-YA9* were 100-fold and 40-fold upregulated, respectively (Fig. S9).

**Fig. 7:**
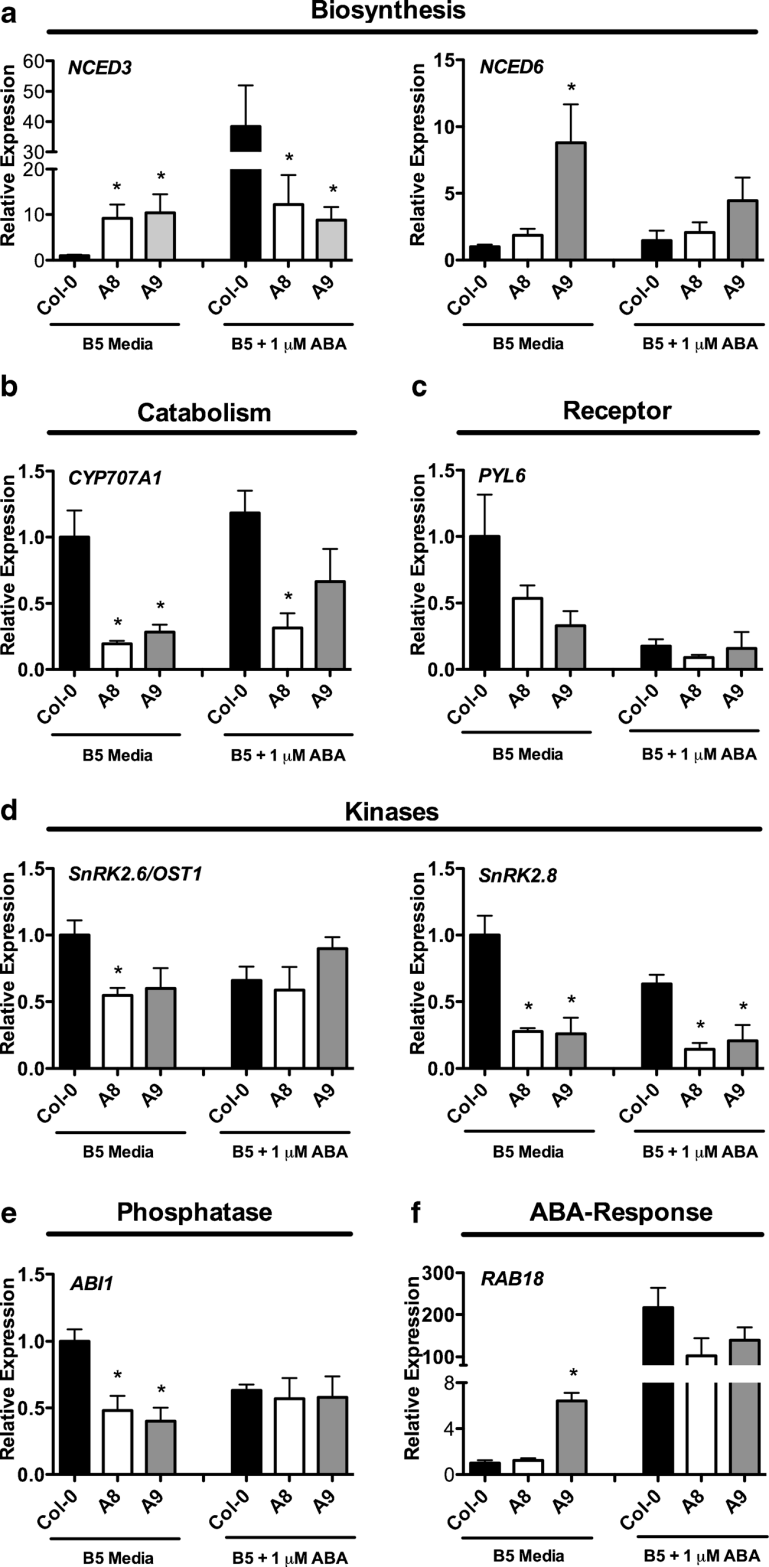
ABA response genes are misregulated in *p35S::NF-YA8* and *p35S::NF-YA9* seeds. Gene expression analyzed by qPCR for genes involved in ABA **a** biosynthesis, **b** catabolism, **c** receptors, **d** kinases, **e** phosphatases, and **f** responses. *Asterisks* represent significant differences derived from two-way ANOVA (*p* < 0.05), in which genotype and seed growth media are the two variables, followed by Bonferroni multiple comparisons post hoc test against Col-0 on B5 media or on B5+1 μM ABA

Initially, the expression of ABA-related markers on seeds incubated on B5 media was examined. Two members of the 9-*cis*-epoxycarotenoid dioxygenase (*NCED*) gene family, *NCED3* and *NCED6*, were misregulated in the overexpressors (Fig. 7a). These genes control the rate-limiting step of ABA biosynthesis during dormancy and seed germination (Ruggiero et al. 2004; Lefebvre et al. 2006; Frey et al. 2012). *NCED3* was significantly upregulated in both *p35S:: NF-YA8* and *p35S::NF-YA9* seeds and *NCED6* in *p35S::NF-YA9* seeds. Following synthesis, ABA 8′-hydroxylation is a key mechanism by which ABA is catabolized. A *CYP707A* gene family member that encodes ABA 8′-hydroxylases during dormancy and seed germination, *CYP707A1* (Okamoto et al. 2006), was significantly downregulated in both *p35S:: NF-YA8* and *p35S::NF-YA9* seeds (Fig. 7b). ABA signaling components were also misregulated in the overexpressors, including the *PYL6* ABA receptor, the *SnRK2.6/OST1* and *SnRK2.8* kinases, and the *ABI1* phosphatase (Fig. 7c–e). In addition, *RAB18*, a well-known ABA response gene (Lang and Palva 1992), was sixfold upregulated in *p35S::NF-YA9* seeds (Fig. 7f).

Expression of these genes after ABA treatment was also evaluated. Strong upregulation (216-fold) of *RAB18* in parental Col-0 showed that the ABA treatment was successful (Fig. 7f). When the seeds where incubated on ABA, *NCED3* was 40-fold upregulated in response to ABA in the wild type but only 10-fold upregulated in the overexpressors (Fig. 7a). ABA marker genes, *ABF3*, *AIA*, and *HAB1*, showed a similar trend (Fig. S10). In addition, *CYP707A1* was significantly downregulated in *p35S::NF-YA8* seeds compared to the wild type (Fig. 7b) and *SnRK2.8* was twofold downregulated in wild type and nearly 10-fold downregulated in the overexpressors on ABA (Fig. 7d).

## Discussion

In the presence of ABA, *p35S::NF-YA*-expressing seeds can show opposing germination phenotypes. Overexpressors of *NF-YA1* and *NF-YA9* were hyposensitive, whereas overexpressors of *NF-YA2*, *NF-YA4*, *NF-YA5*, *NF-YA7*, *NF-YA8*, and *NF-YA10* were hypersensitive to ABA. Opposing germination phenotypes were previously observed for the *NF-YC* subunits. An *nf-yc3 nf-yc9* double mutant and an *nf-yc3 nf-yc4 nf-yc9* triple mutant showed reduced germination inhibition in response to ABA, whereas single and double mutants with *nf-yc4* showed hypersensitivity to ABA (Kumimoto et al. 2013). It is important to note that in the case of *nf-yc* observations, these were based on loss-of-function/hypomorphic mutations. Thus, opposing phenotypes are not necessarily a simple artifact of ectopic overexpression. While opposing germination phenotypes have not been published on the *NF-YB* subunits, the overexpression of two paralogs in the *NF-YB* family, *NF-YB2* and *NF-YB3*, led to ABA hypersensitivity (Kumimoto et al. 2013). The presence of opposing ABA phenotypes in *NF-YA* and *NF-YC* subunits and the fact that NF-Y complexes bind DNA as a trimer (Sinha et al. 1996; Romier et al. 2003; Nardini et al. 2013) suggests that a similar phenomenon would be expected with the *NF-YB* subunits. Supporting this hypothesis, preliminary data from the Holt Lab suggests that overexpression of some *NF-YB*s also leads to ABA hyposensitivity (BFH, unpublished data). These results indicate that while the NF-Y proteins have retained high degrees of similarity, especially in the residues necessary for NF-Y complex formation and DNA binding (Siefers et al. 2009), they may be evolving unique, even antagonistic, regulatory roles for some processes. Similar phenomena from plant transcription factor families include the auxin response factors (ARFs), which include both activators and repressors of auxin response elements (Ulmasov et al. 1999) and WRKY family members, which include both positive and negative regulators of disease resistance (Eulgem and Somssich 2007).

*NF-YC3* and *NF-YC9*, the two *NF-YC* subunits with mutants hyposensitive to ABA, are paralogs whereas *NF-YC4* (mutant hypersensitive to ABA) is more distantly related (Siefers et al. 2009). Similarly, ABA responses for the *NF-YA*s appear connected to their phylogenetic relationships. The two *NF-YA* subunits that are hyposensitive to ABA during seed germination, *NF-YA1* and *NF-YA9*, are closely related paralogs sharing a recent common ancestor (Fig. 1a). Similarly, *NF-YA2* and *NF-YA10* also share a recent common ancestor and both are hypersensitive to ABA. While amino acid alignments in the conserved domains of all 10 NF-YA are highly similar, NF-YA1 and NF-YA9 do have a few unique amino acids that will provide targets for future mutational analyses towards uncovering the specific changes leading to functional differences.

Our findings of reduced ABA sensitivity in seeds overexpressing *NF-YA1* and *NF-YA9* are in contrast to a recent report by Mu et al. (2013) where they reported hypersensitivity. However, the authors appear to define germination as emerged plants after 5 days (i.e., visible cotyledons on a growth plate)—what might be more properly defined as the “greening rate” (Kim et al. 2004). Here, germination is more narrowly defined as the emergence of the radical from the seed coat (Bewley 1997). This is an important distinction as previous research suggests that these two phenotypes are not always directly correlated (Kim et al. 2004; Kumimoto et al. 2013). In fact, *nf-yc9* single mutants did not have a germination phenotype but showed an early greening phenotype. Further, in contrast to an ABA-hypersensitive germination phenotype, *nf-yc4 nf-yc9* double mutants also had an early greening phenotype (Kumimoto et al. 2013). If the same 5-day time point is examined in isolation for the current data, it is in agreement with that reported by Mu et al. (2013) for *NF-YA1* overexpression. However, this hides the fact that most of the *NF-YA1* overexpressors germinate significantly faster than parental Col-0 (Fig. 5a). The day 5 (and later) measurement for *NF-YA1* suggests that total germination percentage never reaches 100 %, but, nevertheless, those that do germinate do so more quickly than Col-0. Thus, our data and previous data strongly suggest that germination and greening are separable processes that need to be carefully defined and quantified as such. Further, although *NF-YA9* overexpression led to reduced ABA sensitivity during germination, the seedlings were hypersensitive to ABA during root elongation. This demonstrates that ABA sensitivity can vary significantly at different developmental time points. The ABRE-binding bZIP transcription factor ABF2 also shows a similar phenomenon (Kim et al. 2004). While *p35S::ABF2* seeds germinate as wild type on ABA, they are hypersensitive to ABA during root growth. In contrast, overexpressing *ABF3* and *ABF4* (members of the same subfamily) results in hypersensitivity to ABA during both seed germination and root elongation (Kang et al. 2002).

It is possible that the NF-Y both physically interacts with and regulates the expression of genes that mediate seed germination in response to ABA. In the case of physical interactions, it was shown that NF-YB and NF-YC subunits physically interact with transcription factors that mediate ABA responses, including ABFs, HY5, and bZIP67 (Yamamoto et al. 2009; Kumimoto et al. 2013). In the current study, *NCED3* and *NCED6*, genes that regulate the rate-limiting step of ABA biosynthesis during germination (Ruggiero et al. 2004; Lefebvre et al. 2006; Frey et al. 2012), were upregulated and *CYP707A1*, a gene that regulates ABA catabolism during germination (Okamoto et al. 2006), was downregulated. It is possible that *NF-YA* regulates the level of ABA during germination and that the overexpression of *NF-YA* genes led to higher levels of ABA in seeds due to increased production and decreased breakdown. In addition to genes that regulate ABA biosynthesis and catabolism, genes that regulate ABA signaling were downregulated. The downregulation of ABA signaling genes is consistent with a previous publication, which showed similar results with *NF-YA2*, *NF-YA3*, *NF-YA7*, and *NF-YA10* overexpressors (Leyva-Gonzalez et al. 2012). This shows that most members of the *NF-YA* family are able to regulate ABA signaling components during germination and other ABA-mediated developmental responses. In addition to misregulation of ABA-related markers, ABA-induced genes showed attenuated response to ABA application. Similarly, ABA induction of known ABA-induced genes was reduced or eliminated in the *pyr1 pyl1 pyl2 pyl4 pyl5 pyl8* sextuple mutant (Gonzalez-Guzman et al. 2012) and the *snrk2.2 snrk2.3 snrk2.6* triple mutant (Fujii and Zhu 2009; Fujita et al. 2009; Nakashima et al. 2009). The current study and Leyva-Gonzalez et al. (2012) have shown the downregulation of ABA receptors and kinases in *NF-YA* overexpressors. It is possible that the reduced induction of ABA-regulated genes in the *NF-YA* overexpressors is partly due to the downregulation of the receptors and kinases. Although this study identified opposing ABA phenotypes in the *NF-YA* family during germination, the qPCR analysis did not identify opposingly regulated genes. A high-throughput analysis using microarray or RNA-seq techniques that identifies global changes in gene expression may be required to identify the genes that lead to the opposing germination phenotypes.

Our finding that *p35S::NF-YA5* is resistant to abiotic stress is in agreement with that of Li et al. (2008) where the authors demonstrate that *NF-YA5* overexpressors are drought tolerant. The authors found that *NF-YA5* transcript was strongly induced by drought conditions. A miRNA, *miR169*, which targets the *NF-YA5* transcript, was downregulated during drought conditions and ABA treatment, and this decrease in *miR169* was partially responsible for the increase in *NF-YA5* transcript accumulation. Similar examples are seen in plant species such as *Medicago truncatula*, soybean (*Glycine max*), and aspen (*Populus tremuloides*), where *miR169* is demonstrated to regulate *NF-YA* transcripts during diverse development programs such as nodulation, drought responses, and vegetative bud formation (Combier et al. 2006; Ni et al. 2013; Potkar et al. 2013). Because most *NF-YA* genes are predicted targets of *miR169* (Rhoades et al. 2002), their role in ABA-mediated germination and embryo development needs further investigation.

Members of the *NF-Y* gene family, common to all eukaryotes, have undergone a large expansion in the plant kingdom; however, the significance of this expansion is not well understood. The same *NF-YC* family members that have opposing roles during germination actually work together to regulate flowering time (Kumimoto et al. 2010, 2013), demonstrating both unique and overlapping roles for *NF-Y* during plant development. The current study adds to the growing evidence for both unique and overlapping roles for the *NF-Y* in the plant lineage by identifying the opposing role for the *NF-YA* family during germination. It is important to note that studying the complete gene family aided in identifying the opposing roles for this genes family. Future studies of *NF-YA* family roles during development can potentially identify similar unique and overlapping responses and may eventually help explain the evolutionary advantages for the expansion of the plant *NF-Y*s.

## Electronic supplementary material

Below is the link to the electronic supplementary material.

ESM 1 (PDF 2365 kb)
