# Supplementary material for: Gene Family Analysis of the Arabidopsis NF-YA Transcription Factors Reveals Opposing Abscisic Acid Responses During Seed Germination
Source: Plant Mol Biol Report. 2014 Feb 25;32(5):971–86. doi: 10.1007/s11105-014-0704-6 (PMC4149875; doi:10.1007/s11105-014-0704-6)
Supplement: Supplementary file 1 — (PDF 2365 kb) [file 11105_2014_704_MOESM1_ESM.pdf]

Supplementary data for

Overexpression of the *Arabidopsis* NF-YA transcription factor family leads to opposing abscisic acid responses during seed germination

Chamindika L. Siriwardana, Roderick W. Kumimoto, Daniel Jones, and *Ben F. Holt III

*Corresponding author. e-mail. benholt@ou.edu

This file includes:

Table S1 List of homozygous and hemizygous *p35S::NF-YA* plant lines.
Fig. S1 Multiple sequence alignment of the Arabidopsis NF-YA.
Fig. S2 Identity matrix for the Arabidopsis NF-YA.
Fig. S3 Quantification of growth defects in *p35S::NF-YA* plant lines.
Fig. S4 Characterization of cotyledon-like leaves in *p35S::NF-YA5*.
Fig. S5 Abiotic stress responses of *p35S::NF-YA5*.
Fig. S6 Confirmation of nuclear localization for NF-YA proteins.
Fig. S7 ABA responses during root elongation.
Fig. S8 Publically available microarray data for the expression of *NF-YA* genes in seeds.
Fig. S9 *NF-YA8* and *NF-YA9* expression in *p35S::NF-YA8* and *p35S::NF-YA9* seeds.
Fig. S10 ABA-induction of gene expression in *NF-YA* overexpressors.

Table S1 List of homozygous and hemizygous *p35S::NF-YA* plant lines.

AGI#	Construct	Plant line#	Homozygous or Hemizygous
**At5g12840**	*p35S::NF-YA1-CFP/HA*	1	Homozygous
		2	Homozygous
**At3g05690**	*p35S::NF-YA2-CFP/HA*	1	Homozygous
		2	Homozygous
**At1g72830**	*p35S::NF-YA3-CFP/HA*	1	Homozygous
		2	Homozygous
**At2g34720**	*p35S::NF-YA4-CFP/HA*	1	Homozygous
		2	Hemizygous
**At1g54160**	*p35S::NF-YA5-CFP/HA*	1	Hemizygous
		2	Hemizygous
**At3g14020**	*p35S::NF-YA6-CFP/HA*	1	Hemizygous
		2	Hemizygous
**At1g30500**	*p35S::NF-YA7-CFP/HA*	1	Homozygous
		2	Homozygous
**At1g17590**	*p35S::NF-YA8-CFP/HA*	1	Homozygous
		2	Homozygous
**At3g20910**	*p35S::NF-YA9-CFP/HA*	1	Homozygous
		2	Homozygous
**At5g06510**	*p35S::NF-YA10-CFP/HA*	1	Hemizygous
		2	Homozygous

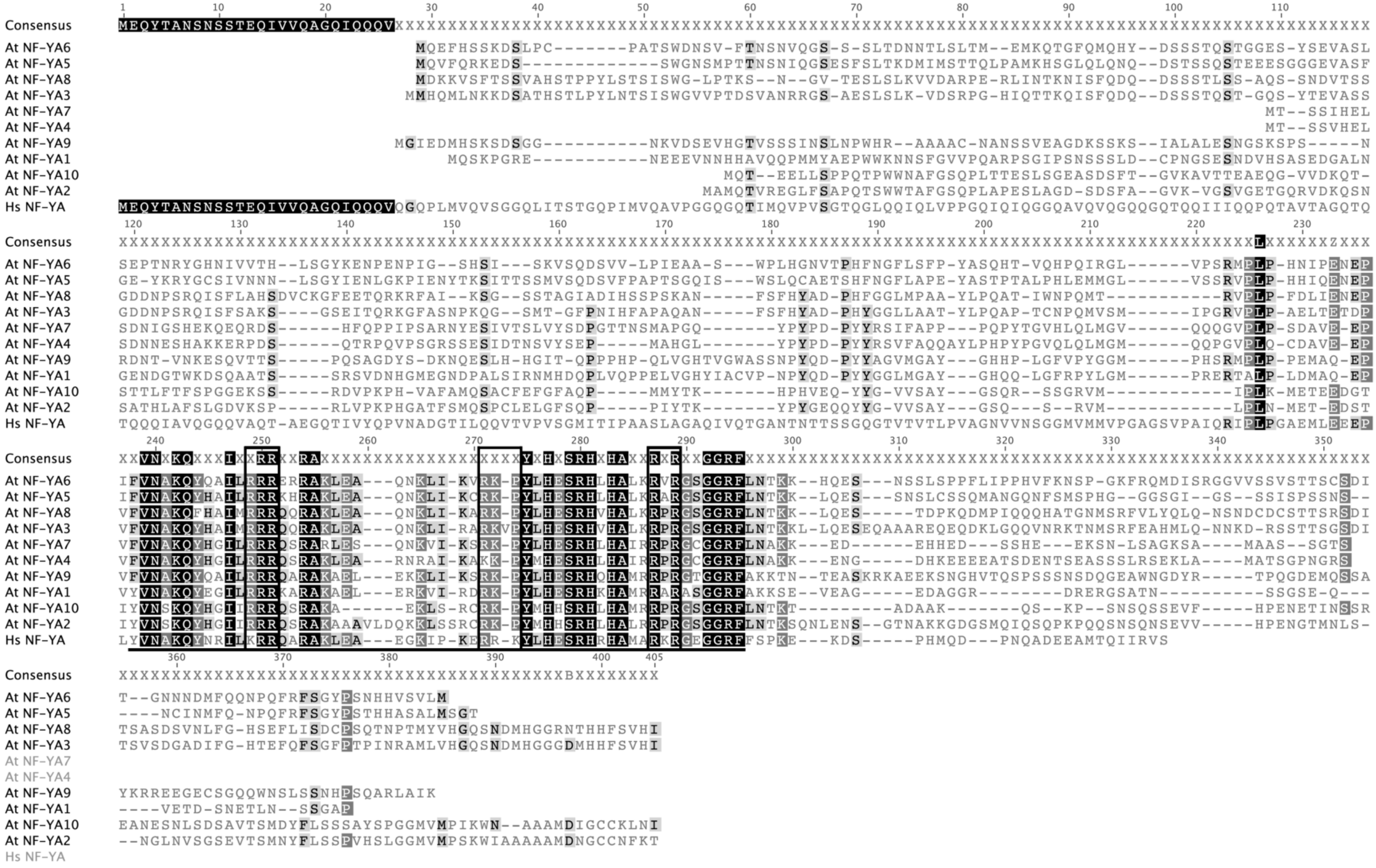

Fig. S1 Multiple sequence alignment of full-length NF-YA protein sequences. At, *Arabidopsis thaliana*; Hs, *Homo sapiens*. The conserved core domain is underlined in black. The three black boxes correspond to nuclear localization signal in human.

a

	AtNF-YA2	AtNF-YA10	AtNF-YA1	AtNF-YA9	AtNF-YA5	AtNF-YA6	AtNF-YA3	AtNF-YA8	AtNF-YA4	AtNF-YA7	HsNF-YA
AtNF-YA2		63.2%	23.0%	24.0%	22.3%	21.4%	21.6%	19.4%	27.7%	26.5%	19.6%
AtNF-YA10	63.2%		21.5%	20.9%	18.8%	18.5%	20.6%	18.5%	27.5%	27.0%	20.2%
AtNF-YA1	23.0%	21.5%		41.6%	22.2%	18.8%	22.4%	20.0%	26.8%	28.6%	20.4%
AtNF-YA9	24.0%	20.9%	41.6%		22.8%	25.0%	23.1%	21.8%	29.8%	31.0%	20.3%
AtNF-YA5	22.3%	18.8%	22.2%	22.8%		56.3%	34.4%	33.7%	30.5%	32.1%	21.3%
AtNF-YA6	21.4%	18.5%	18.8%	25.0%	56.3%		35.9%	33.7%	30.1%	30.6%	18.6%
AtNF-YA3	21.6%	20.6%	22.4%	23.1%	34.4%	35.9%		63.9%	33.3%	33.0%	21.3%
AtNF-YA8	19.4%	18.5%	20.0%	21.8%	33.7%	33.7%	63.9%		32.3%	31.5%	19.7%
AtNF-YA4	27.7%	27.5%	26.8%	29.8%	30.5%	30.1%	33.3%	32.3%		64.5%	23.0%
AtNF-YA7	26.5%	27.0%	28.6%	31.0%	32.1%	30.6%	33.0%	31.5%	64.5%		25.7%
HsNF-YA	19.6%	20.2%	20.4%	20.3%	21.3%	18.6%	21.3%	19.7%	23.0%	25.7%	

b

	AtNF-YA1	AtNF-YA9	AtNF-YA4	AtNF-YA7	AtNF-YA3	AtNF-YA8	AtNF-YA5	AtNF-YA6	AtNF-YA2	AtNF-YA10	HsNF-YA
AtNF-YA1		78.9%	64.9%	70.2%	66.7%	64.9%	70.2%	66.7%	57.4%	63.2%	64.9%
AtNF-YA9	78.9%		71.9%	77.2%	73.7%	75.4%	73.7%	75.4%	59.0%	63.2%	68.4%
AtNF-YA4	64.9%	71.9%		86.0%	74.1%	75.9%	74.1%	72.4%	61.3%	63.8%	63.8%
AtNF-YA7	70.2%	77.2%	86.0%		75.9%	77.6%	77.6%	75.9%	61.3%	63.8%	65.5%
AtNF-YA3	66.7%	73.7%	74.1%	75.9%		93.1%	82.8%	81.0%	64.5%	63.8%	63.8%
AtNF-YA8	64.9%	75.4%	75.9%	77.6%	93.1%		86.2%	84.5%	61.3%	60.3%	65.5%
AtNF-YA5	70.2%	73.7%	74.1%	77.6%	82.8%	86.2%		91.4%	64.5%	63.8%	67.2%
AtNF-YA6	66.7%	75.4%	72.4%	75.9%	81.0%	84.5%	91.4%		61.3%	60.3%	65.5%
AtNF-YA2	57.4%	59.0%	61.3%	61.3%	64.5%	61.3%	64.5%	61.3%		83.9%	51.6%
AtNF-YA10	63.2%	63.2%	63.8%	63.8%	63.8%	60.3%	63.8%	60.3%	83.9%		55.2%
HsNF-YA	64.9%	68.4%	63.8%	65.5%	63.8%	65.5%	67.2%	65.5%	51.6%	55.2%	

Fig. S2 Identity matrix of the NF-YA proteins. a) Full-length protein. b) Conserved domain. At, *Arabidopsis thaliana*; Hs, *Homo sapiens*. Note that pairs of Arabidopsis NF-YA paralogs demonstrate high identity across the full-length protein sequence and the conserved domain.

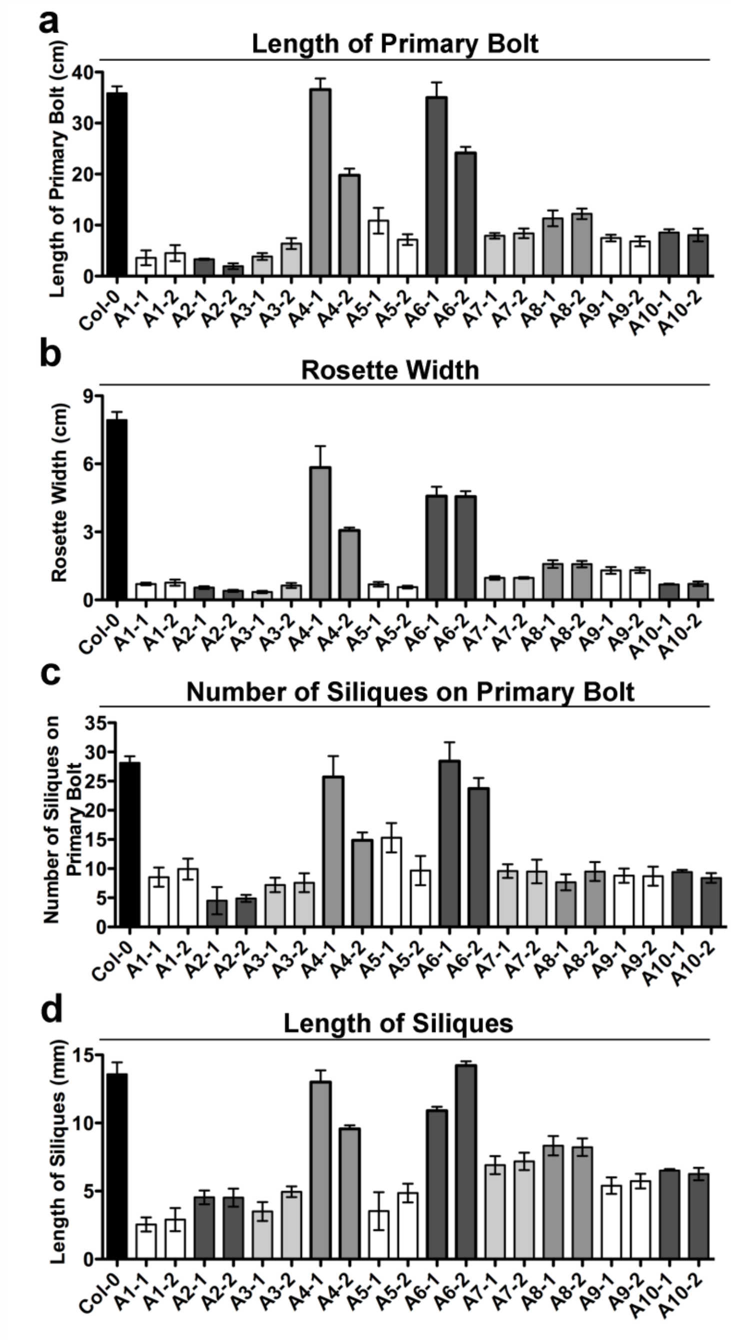

Fig. S3 Overexpression of the *NF-YA* gene family leads to growth defects. Quantitative measurements of (a) plant height (b) rosette width (c) number of siliques on primary bolt (d) length of siliques.

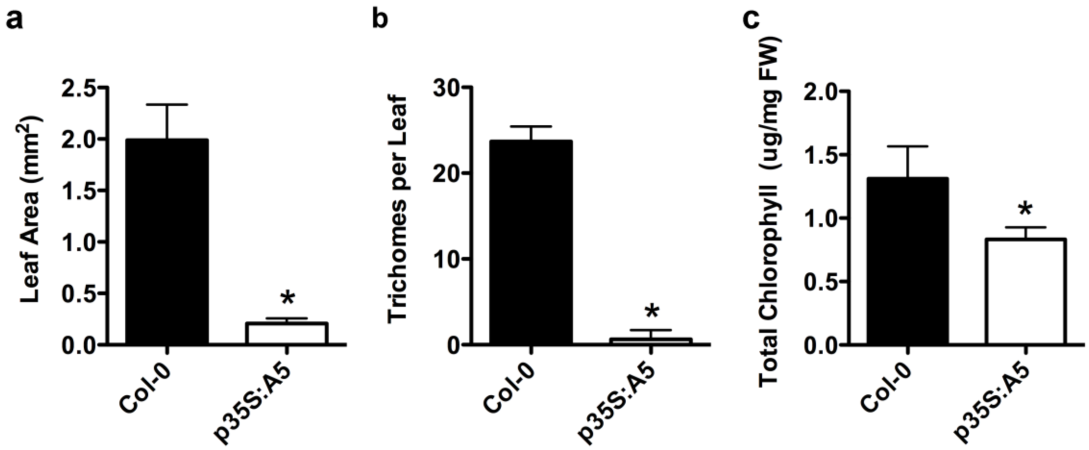

Fig. S4 Quantifications of the cotyledon-like leaves in *p35S::NF-YA5*. (a) Leaf area (b) number of trichomes (c) chlorophyll content. Asterisks represent significant differences derived from Student's t-tests.

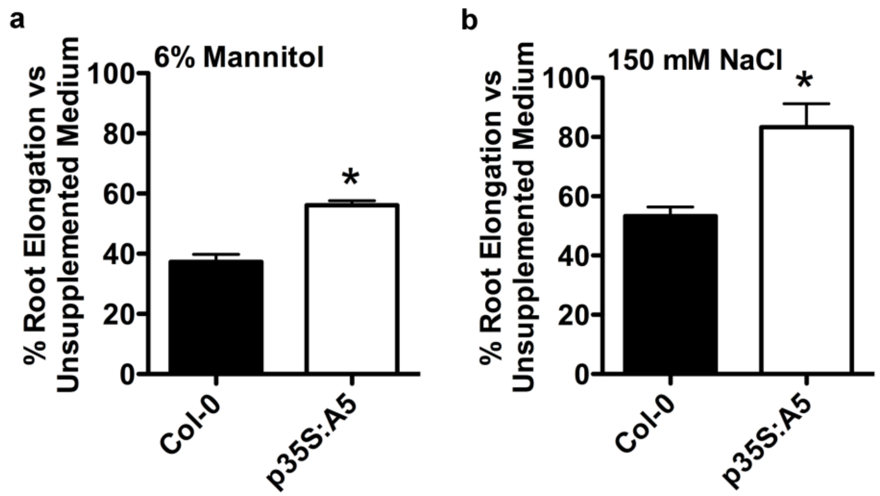

Fig S5. Abiotic stress responses of *p35S::NF-YA5* seedlings. Response to a) 6% mannitol b) 150 mM NaCl. Asterisks represent significant differences derived from Student's t-tests.

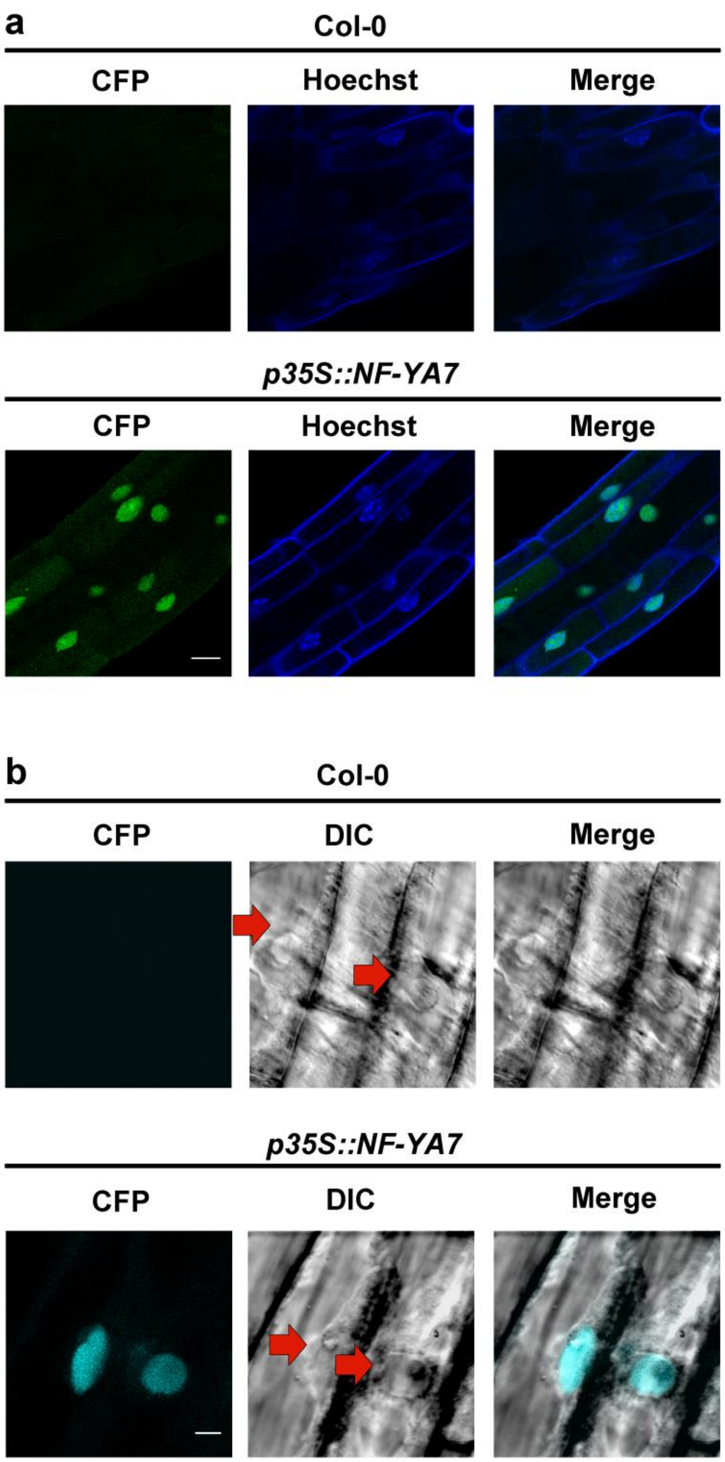

Fig. S6 Confirmation of nuclear localization of NF-YA proteins. (a) Hoechst 33342 (blue) labels nuclei and CFP signal (green) shows localization of NF-YA7. Merged images demonstrate co-localization of DNA label and CFP fluorescence. Scale - 15 µm. (b) Differential Interference Contrast (DIC) imaging (grey) of root cell nuclei (red arrows point to the nuclei) with CFP signal (cyan) showing localization of NF-YA7. Merged images indicate CFP fluorescence is localized to the root cell nuclei. Scale - 5 µm.

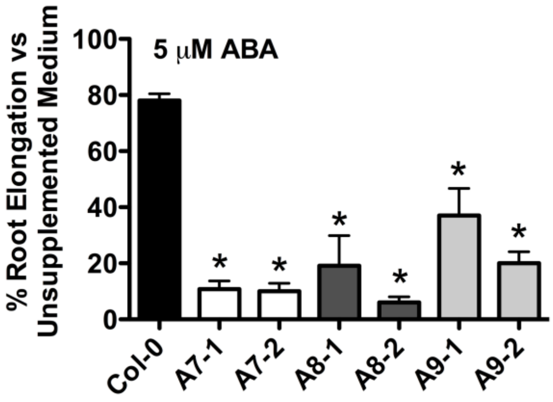

Fig. S7 Overexpression of select *NF-YA* genes leads to ABA hypersensitivity during root elongation. The percent root elongation in growth medium with ABA was compared to the unsupplemented medium for Col-0, *p35S::NF-YA7*, *p35S::NF-YA8*, and *p35S::NF-YA9*. Asterisks represent significant differences derived from one-way ANOVA ($P < 0.05$) followed by Dunnett's multiple comparison post hoc test against Col-0

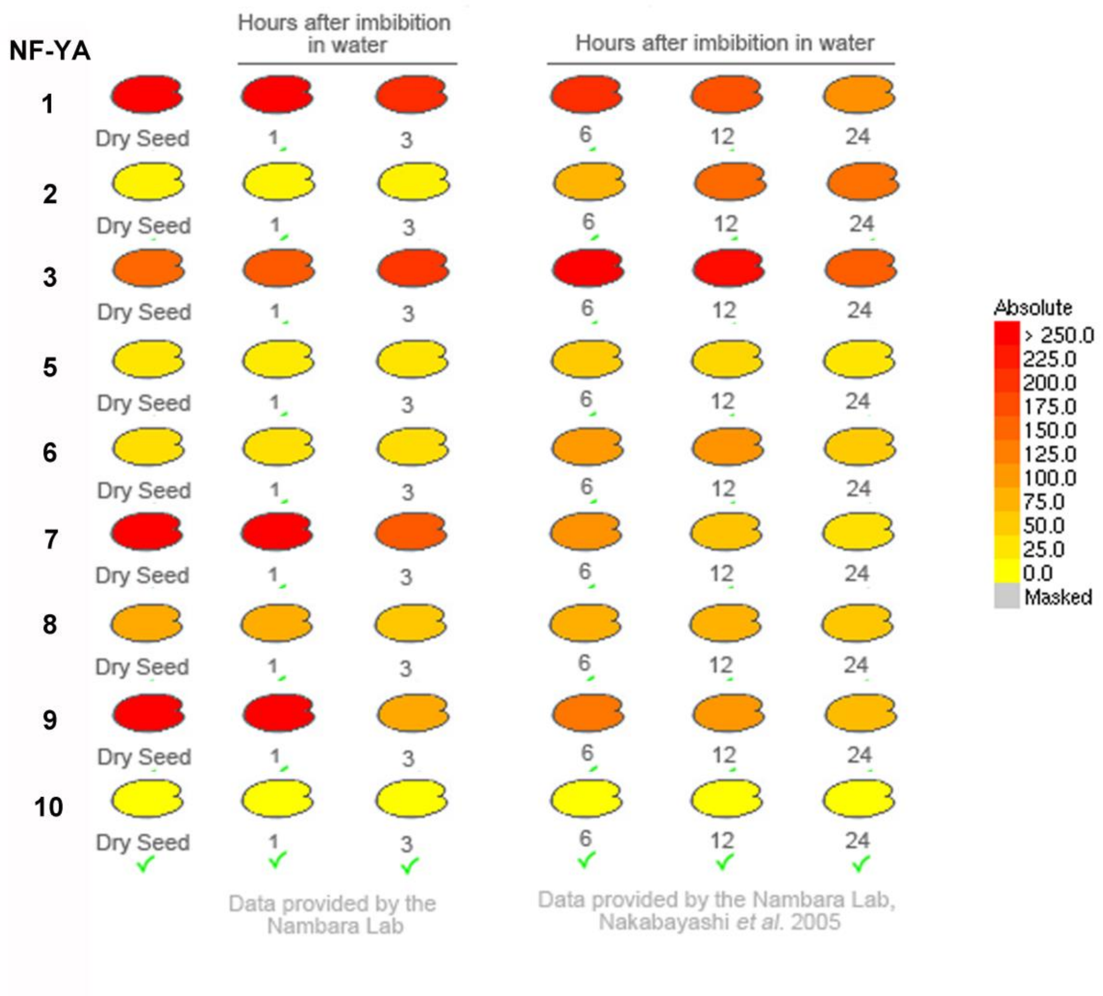

Fig. S8 Publically available microarray data demonstrate that *NF-YA* genes are expressed in seeds. Data was obtained from the Arabidopsis e-FP browser at http://bar.utoronto.ca/efp/cgi-bin/efpWeb.cgi.

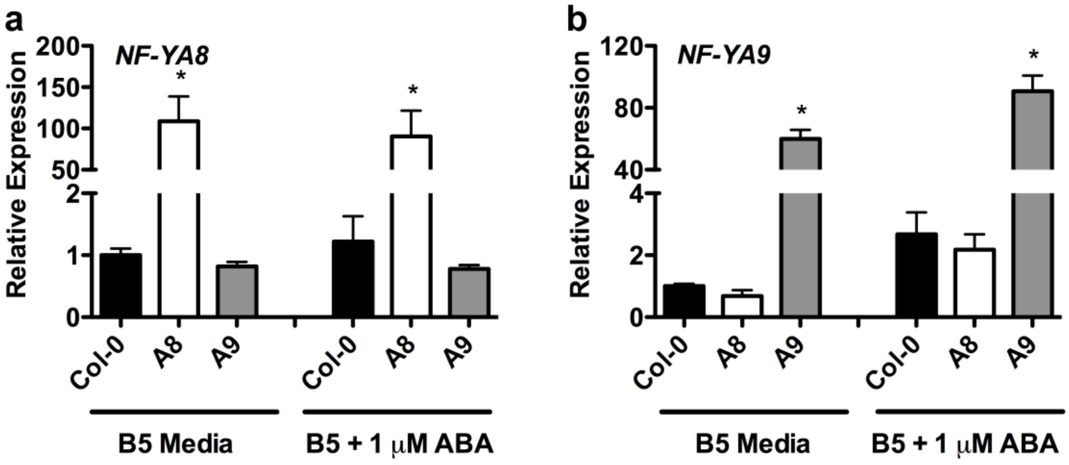

Fig. S9 qPCR analysis of gene expression in *p35S::NF-YA8* and *p35S::NF-YA9* seeds. (A) *NF-YA8* (B) *NF-YA9*. Asterisks represent significant differences derived from two-way ANOVA ($p<0.05$), in which genotype and seed growth media are the two variables, followed by Bonferroni multiple comparisons post hoc test against Col-0 on B5 media or on B5 + 1 μM ABA.

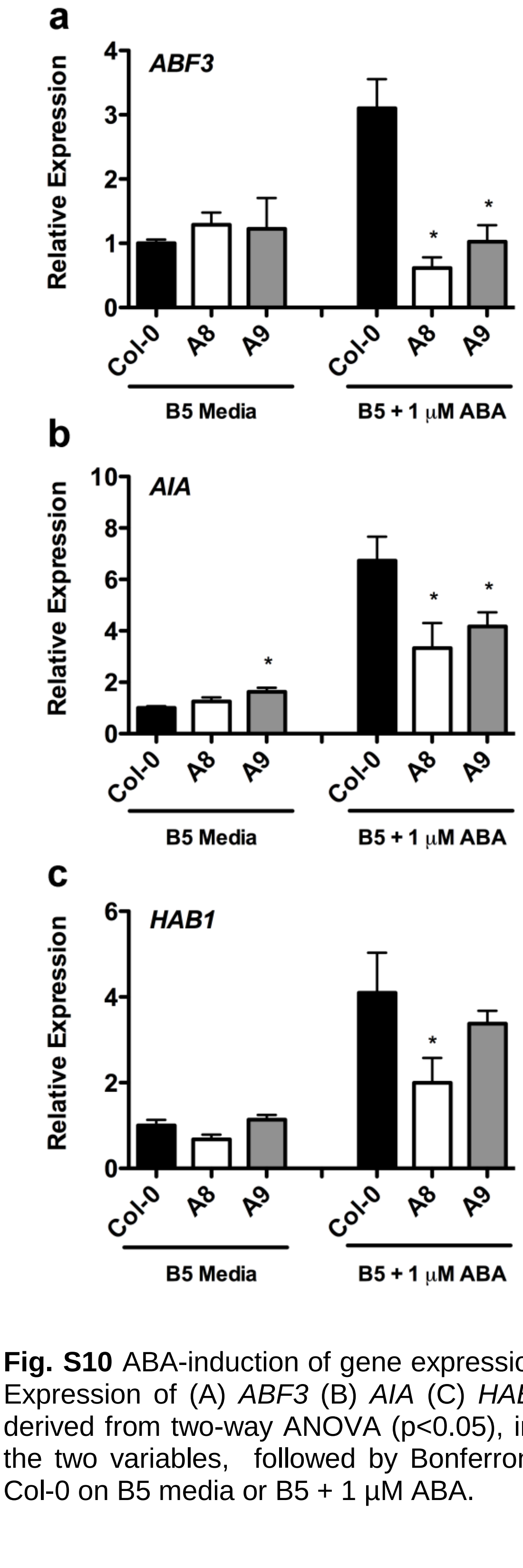

Fig. S10 ABA-induction of gene expression in *p35S::NF-YA8* and *p35S::NF-YA9* seeds. Expression of (A) *ABF3* (B) *AIA* (C) *HAB1*. Asterisks represent significant differences derived from two-way ANOVA ($p<0.05$), in which genotype and seed growth media are the two variables, followed by Bonferroni multiple comparisons post hoc test against Col-0 on B5 media or B5 + 1 μM ABA.
